# Bayesian, Maximum Parsimony and UPGMA Models for Inferring the Phylogenies of Antelopes Using Mitochondrial Markers

**DOI:** 10.4137/ebo.s934

**Published:** 2008-10-06

**Authors:** Haseeb A. Khan, Ibrahim A. Arif, Ali H. Bahkali, Ahmad H. Al Farhan, Ali A. Al Homaidan

**Affiliations:** Molecular Fingerprinting and Biodiversity Unit, Prince Sultan Research Chair Program in Environment and Wildlife, College of Science, King Saud University, Riyadh, Saudi Arabia

**Keywords:** antelopes, mitochondrial DNA, phylogenetic trees, bioinformatics, Bayesian, maximum parsimony, UPGMA

## Abstract

This investigation was aimed to compare the inference of antelope phylogenies resulting from the 16S rRNA, cytochrome-b (cyt-b) and d-loop segments of mitochondrial DNA using three different computational models including Bayesian (BA), maximum parsimony (MP) and unweighted pair group method with arithmetic mean (UPGMA). The respective nucleotide sequences of three Oryx species (*Oryx leucoryx*, *Oryx dammah* and *Oryx gazella*) and an out-group (*Addax nasomaculatus*) were aligned and subjected to BA, MP and UPGMA models for comparing the topologies of respective phylogenetic trees. The 16S rRNA region possessed the highest frequency of conserved sequences (97.65%) followed by cyt-b (94.22%) and d-loop (87.29%). There were few transitions (2.35%) and none transversions in 16S rRNA as compared to cyt-b (5.61% transitions and 0.17% transversions) and d-loop (11.57% transitions and 1.14% transversions) while comparing the four taxa. All the three mitochondrial segments clearly differentiated the genus Addax from Oryx using the BA or UPGMA models. The topologies of all the gamma-corrected Bayesian trees were identical irrespective of the marker type. The UPGMA trees resulting from 16S rRNA and d-loop sequences were also identical (*Oryx dammah* grouped with *Oryx leucoryx*) to Bayesian trees except that the UPGMA tree based on cyt-b showed a slightly different phylogeny (*Oryx dammah* grouped with *Oryx gazella*) with a low bootstrap support. However, the MP model failed to differentiate the genus Addax from Oryx. These findings demonstrate the efficiency and robustness of BA and UPGMA methods for phylogenetic analysis of antelopes using mitochondrial markers.

## Introduction

The antelope Arabian Oryx was extirpated from the wild as a result of massive hunting during early 1970s (Henderson, 1974). Fortunately, the efforts of captive breeding programs succeeded to preserve the Arabian Oryx, which was later reintroduced in certain protected areas (Spalton et al. 1999; Ostrowski et al. 1998; Mesochina et al. 2003). Recently, Iyengar et al. (2007) have recommended maintaining a global perspective for the captive genetic management of Oryx. Individuals from various management programs and regions need to be effectively utilized for sustained future captive breeding in order to ensure that the vital remnants of genetic diversity are retained and represented in future reintroduction programs (Iyengar et al. 2007). It has been suggested that molecular methods can significantly contribute to the captive breeding and reintroduction strategies for conservation of various endangered animals such as Oryx antelopes (Russello and Amato, 2007).

Molecular fingerprinting based on the nucleotide sequence analysis of various mitochondrial genes plays an important role in studying evolutionary relationship among various taxa. Besides its exclusive maternal inheritance and lack of recombination, different segments of mitochondrial DNA (mtDNA) also possess unique features such as conservativeness in protein-coding regions and high variability in non-coding sequences (Ingman et al. 2000; Olivo et al. 1983). Consequently, the evolutionary rate of mtDNA tends to be variable for different regions and has been utilized to examine various levels of phylogenetic relationships. The 12S rRNA gene sequences being highly conserved, are applied to illustrate higher levels of phylogenies (phyla or subphyla) whereas the 16s rRNA sequences are mainly used for phylogenetic studies at mid-categorical levels (families or genera) (Gerber et al. 2001). Since the mitochondrial protein-coding genes and the d-loop evolve comparatively faster they are considered as powerful tools for inferring evolutionary history in mid to lower categorical levels such as genera and species.

Probabilistic modeling of sequence evolution has now become inevitable in phylogenetic inference (Felsenstein, 2001). Despite a positive impact of statistical revolution, the emergence of sophisticated evolutionary models has placed a burden on researchers to select the model most appropriate for their data. It is intriguing that the bioinformatics tool used for phylogenetic analysis may have some influence on the topologies of the resulting trees. An inappropriate choice of evolutionary model can affect the outcome of any phylogenetic analysis by incorrectly estimating tree topologies (Penny et al. 1994; Bruno and Halpern, 1999). Bayesian (BA), maximum likelihood (ML) or unweighted pair group method with arithmetic mean (UPGMA) and maximum parsimony (MP) are the main phylogenetic approaches that are often used side by side. While the choice between them has been contentious at times, they frequently give similar results and if they don’t, they can complement each other (Liberles, 2005). In this investigation, we have compared BA, MP and UPGMA methods for phylogenetic analysis of Oryx antelopes using 16S rRNA, cytochrome-b (cyt-b) and d-loop sequences of mtDNA.

## Methods

The sequences of 16S rRNA, cyt-b and d-loop of the three Oryx species including Arabian Oryx (*Oryx leucoryx*), Scimitar Horned Oryx (*Oryx dammah*) and Plains Oryx (*Oryx gazella*) were obtained from GenBank. The respective sequences of Addax (*Addax nasomaculatus*) were used as outgroup due to its close relationship to Oryx yet representing a separate sister taxa (Hassanin and Douzery, 1999; Iyengar et al. 2006). The Gene-Bank accession numbers and the number of nucleotides for the partial sequences of 16S rRNA, cyt-b and d-loop of the four taxa are: *Oryx leucoryx* (U87021, 342; AF036286, 1143; AJ235326, 1253), *Oryx dammah* (U87020, 342; AJ222685, 1143; AJ235324, 1261), *Oryx gazella* (U87022, 342; AF249973, 1140; AJ235325, 1237) and *Addax nasomaculatus* (U87023, 342; AF034722, 1143; AJ235310, 1324) respectively.

Tajima test statistics (Tajima, 1989) and the test of homogeneity of substitution patterns between sequences were performed after sequence alignments, using MEGA4 software (Tamura et al. 2007) while all the positions containing gaps and missing data were eliminated from the dataset (complete deletion option). The probability of rejecting the null hypothesis that sequences have evolved with the same pattern of substitution was judged from the extent of differences in the base composition biases between sequences (disparity index test) whereas a Monte Carlo test (1000 replicates) was used to estimate the respective *P*-values (Kumar and Gadagkar, 2001).

The sequence data were subjected to three different methods of phylogenetic reconstruction: (i) Bayesian (BA), (ii) unweighted pair group method with arithmetic mean (UPGMA) and (iii) maximum parsimony (MP). The gamma-corrected Bayesian inference of phylogeny was conduced using MrBayes software (Huelsenbeck and Ronquist, 2001) and the Bayesian trees were visualized with TreeView software (Page, 1996). For UPGMA method, the phylogenetic analyses were performed using the evolutionary distances computed by maximum composite likelihood method (Sneath and Sokal, 1973; Tamura et al. 2004). For MP method, the maximum parsimonious trees were obtained using the close-neighbor-interchange algorithm in which the initial trees were obtained with the random addition of sequences for 10 replicates (Eck and Davhoff, 1966; Nei and Kumar, 2000). Both UPGMA and MP analyses were performed using MEGA4 software and the bootstrap consensus trees inferred from 1000 replicates were taken to represent the evolutionary history of the taxa analyzed (Felsenstein, 1985; Tamura et al. 2007).

## Results

**Figure 1:**
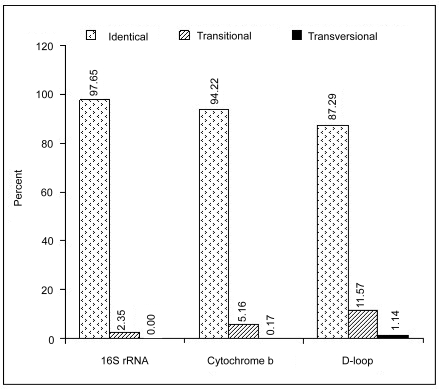
Average frequencies of identical (conserved) and substituted (transitional and transversional) sites observed in sequence comparison for various segments of mtDNA.

Both 16S rRNA and cyt-b sequences were perfectly aligned without any insertions/deletions (indels) whereas numerous indels at various sites of different taxa were required to align the sequences of d-loop (please refer to electronic supplementary file). The average frequencies of identical (conserved) sequences between the taxa were 97.65% for 16S rRNA, 94.22% for cyt-b and 87.29% for d-loop (Fig. 1). On an average there were few transitions (2.35%) and none transversions in 16S rRNA as compared to cyt-b (5.61% transitions and 0.17% transversions) and d-loop (11.57% transitions and 1.14% transversions) (Fig. 1).

The results of Tajima’s neutrality are given in Table 1. Both the number of segregating sites (S) and nucleotide diversities (π) were directly correlated and were in the order of 16S rRNA (S = 17, π = 0.025) < cyt-b (S = 125, π = 0.058) < d-loop (S = 270, π = 0.122) (Table 1). The test of homogeneity of substitution patterns showed certain identities and certain variations in disparity index as well as Monte Carlo probability for different mitochondrial markers (Table 2).

**Figure 2:**
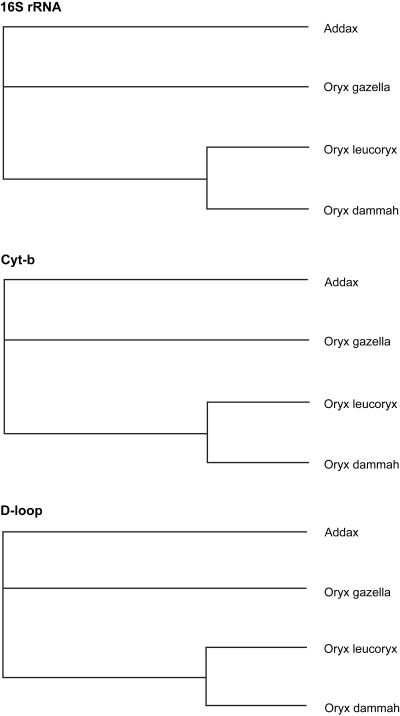
Bayesian method for inferring phylogenetic relationship among various Oryx species using Addax as an outgroup.

**Figure 3:**
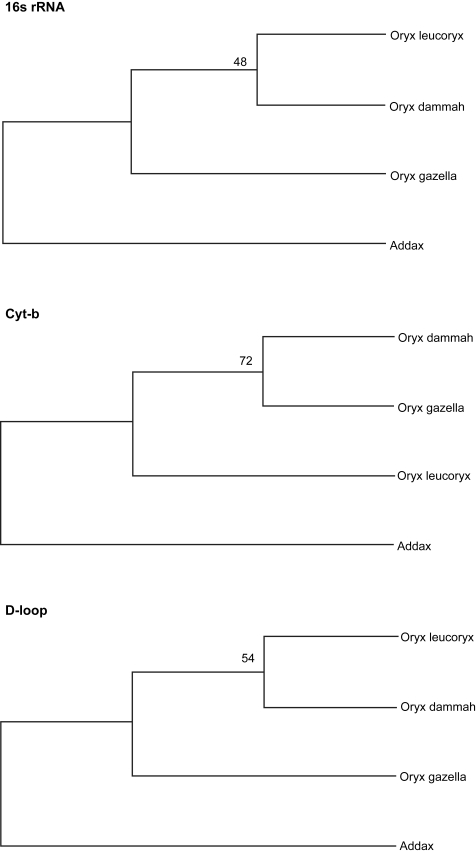
UPGMA method for inferring phylogenetic relationship among various Oryx species using Addax as an outgroup. The bootstrap consensus trees inferred from 1000 replicates are taken to represent the phylogeny. The evolutionary distances were computed using the maximum composite likelihood method.

**Figure 4:**
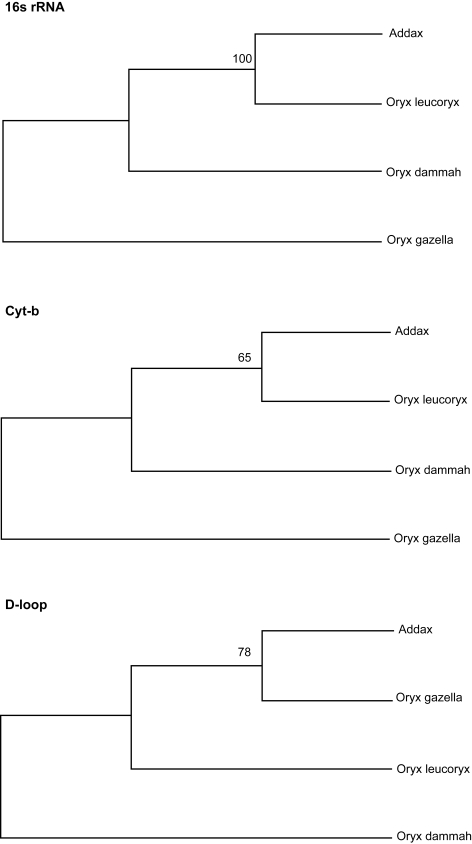
Maximum parsimony method for inferring phylogenetic relationship among various Oryx species using Addax as an outgroup. The bootstrap consensus tree inferred from 1000 replicates is taken to represent the evolutionary history of the taxa analyzed. The maximum parsimonious tree was obtained using the close neighbor interchange algorithm in which the initial trees were obtained with the random addition of sequences (10 replicates).

The topologies of all the Bayesian trees were identical irrespective of the marker type, which clearly differentiated the genus Addax from Oryx, and grouped *Oryx dammah* with *Oryx leucoryx* (Fig. 2). The UPGMA trees resulting from 16S rRNA and d-loop sequences were also identical (*Oryx dammah* grouped with *Oryx leucoryx*) to Bayesian trees except that the UPGMA tree based on cyt-b showed a slightly different phylogeny (*Oryx dammah* grouped with *Oryx gazella*) with a low bootstrap support (Fig. 3). The MP method failed to differentiate the genus Addax from Oryx and *Addax nasomaculatus* was either grouped with *Oryx leucoryx* (16S rRNA or cyt-b) or with *Oryx gazella* (d-loop) (Fig. 4).

## Discussion

In conservation genetics, knowledge of the relatedness between individuals is particularly important for captive breeding programs to recover small populations (Frankham et al. 2002; Montgomery et al. 1997). Genetically impoverished endangered populations often fail to exhibit signs of recovery until crossed with individuals from other populations (Land and Lacy, 2000; Westemeier et al. 1998). However, if the strategy is to maintain the genetic identity of the population, the introduced individuals should be closely related to the recipient population. Recently, Masembe et al. (2006) have recommended the need of conservation efforts to preserve genetic identity of various oryx groups. Molecular methods play an important role in estimating the relatedness between individuals by comparing the genotypes at a number of informative loci (Sunnucks, 2000). The high mutation rate of mtDNA compared to nuclear genes renders mtDNA sequences to possess high levels of informative variation that could be utilized for resolving taxonomic relationship in conservation genetics using appropriate bioinformatics tools.

We observed no indels in 16S rRNA and cyt-b genes whereas numerous indels were noticed in the aligned sequences of d-loop which is in agreement with an earlier study reporting specific indels in the d-loop of Oryx species (Iyengar et al. 2006). The frequency of conserved sequences was highest in 16S rRNA gene followed by cyt-b and was lowest in d-loop region whereas the converse was true for the substitutions (Fig. 1). Most of the substitutions in the mitochondrial regions studied were transitional indicating a recent species history. Factually, transitions are typically observed more often than transversions in the evolution of real sequences.

The BA model with gamma correction appears to be the most efficient method as it produced identical trees using the nucleotide sequences of any of the three segments of mtDNA (Fig. 2). Bayesian inference has been successfully applied to inference of phylogenetic trees using mitochondrial and nuclear genes (Doudy et al. 2003; Xiong et al. 2002; Ragan et al. 2003). Although likelihood-based approaches have proven to be especially powerful for inferring phylogenetic trees they tend to be prohibitively slow due to the requirement of multidimensional space for possible outcomes (optimal trees) and the computational complexity of bootstrap repetitions. On the other hand, BA phylogenetic inference holds promise as an alternative to ML, particularly for large molecular-sequence data sets. Moreover, BA phylogenetic inference has been shown to be as or more robust to ML, particularly when among-sites rate variation is modeled using a gamma distribution (Mar et al. 2005).

The UPGMA model also produced similar phylogenies to BA model for 16S rRNA and d-loop sequences however cyt-b inferred a different phylogeny (Fig. 3). These differential phylogenies may be associated with comparatively high variations in non-coding d-loop than coding cyt-b due to reduced functional constraints and relaxed selection pressure. Although, increased polymorphism in d-loop segment may render it superior to cyt-b for species or sub-species level identification, the possibility of reduced phylogenic information due to back mutations and parallel substitutions in rapidly-evolving d-loop may not be ruled out. It is also important to mention that changing the outgroup species or the length of d-loop segment can significantly alter the topology of phylogenic trees (Iyenger et al. 2006).

The MP model resulted different phylogenetic inferences than those from the BA and UPGMA models (Fig. 4). A certain degree of contradictive phylogeny using mitochondrial markers has been noticed earlier (Jogger and Garrido, 2001). Shoup and Lewis (2003) have performed BA analyses as well as ML bootstrapping and revealed several instances of conflict between these two approaches to measuring edge support. Kim et al. (2006) have also observed some variation in the topologies of BA and ML-based phylogenic trees to explain the origin and evolution of coronaviruses.

In conclusion, this bioinformatics approach demonstrates the superiority of BA and UPGMA models over MP model for phylogenetic analysis using different regions of mtDNA or other datasets of this size. However, the implication of these findings to different data structures e.g. multiple sequences and more numbers of taxa or outgoups is not clear and needs further investigations.

## Figures and Tables

**Table 1:** Tajima’s neutrality test for 4 taxa using different mitochondrial markers.

	Number of sites (m)	Number of segregating sites (S)	Ps = S/m	Nucleotide diversity (π)	Tajima test statistics (D)
16S rRNA	4	17	0.049708	0.025341	−0.667112
Cyt-b	4	125	0.109457	0.058085	−0.283933
D-loop	4	270	0.224439	0.122333	−0.007559

**Table 2:** The test of homogeneity of substitution patterns for different mitochondrial markers.

	Addax	Oryx leucoryx	Oryx dammah	Oryx gazella
*16S rRNA*				
Addax	–	0.000	0.000	0.000
Oryx leucoryx	1.000	–	0.018	0.000
Oryx dammah	1.000	0.074	–	0.041
Oryx gazella	1.000	1.000	0.012*	–
*Cyt-b*				
Addax	–	0.000	0.000	0.000
Oryx leucoryx	1.000	–	0.000	0.000
Oryx dammah	1.000	1.000	–	0.000
Oryx gazella	1.000	1.000	1.000	–
*D-loop*				
Addax	–	0.053	0.000	0.114
Oryx leucoryx	0.261	–	0.000	0.000
Oryx dammah	1.000	1.000	–	0.000
Oryx gazella	0.124	1.000	1.000	–

The estimates of the disparity index per site are shown for each sequence pair above the diagonal. The *P* values based on Monte Carlo test (1000 replicates) are shown below the diagonal. *P < 0.05, statistically significant.
